# Impulsivity and cigarette smoking: discounting of monetary and consumable outcomes in current and non-smokers

**DOI:** 10.1007/s00213-014-3597-z

**Published:** 2014-05-13

**Authors:** Jonathan E. Friedel, William B. DeHart, Gregory J. Madden, Amy L. Odum

**Affiliations:** Department of Psychology, Utah State University, 2810 Old Main Hill, Logan, UT 84322 USA

**Keywords:** Delay discounting, Impulsivity, Self-control, Intertemporal choice, Smoking, Drug abuse, Addiction, Cigarette, Nicotine

## Abstract

**Rationale:**

In delay discounting, temporally remote rewards have less value. Cigarette smoking is associated with steeper discounting of delayed money. The generality of this to nonmonetary outcomes, however, is unknown.

**Objectives:**

We sought to determine whether cigarette smokers also show steep discounting of other delayed outcomes.

**Methods:**

Sixty-five participants (32 smokers and 33 non-smokers) completed four delay-discounting tasks, each involving different hypothetical outcomes. In the monetary task, participants indicated their preference for a smaller amount of money available immediately (titrated across trials) and $100 awarded at delays ranging from 1 week to 25 years (tested in blocks). In the three other discounting tasks the larger-later reward was $100 worth of a favorite food, alcoholic drink, or a favorite form of entertainment. All other aspects of these discounting tasks were identical to the monetary discounting task.

**Results:**

As previously shown, smokers discounted delayed money more steeply than non-smokers did. In addition, smokers discounted delayed food and entertainment rewards more steeply than did nonsmokers. A person’s discounting of one outcome was correlated with discounting of other outcomes. Non-smokers discounted money less steeply than all other outcomes; smokers discounted money significantly less than food.

**Conclusions:**

When compared to nonsmokers, cigarette smokers more steeply discount several types of delayed outcomes. This result, together with the finding that cross-commodity discounting rates were correlated within subjects, suggests that delay discounting is a trait that extends across domains.

## Introduction

Cigarette smoking is the leading cause of preventable death in the United States and results in an estimated $167 billion per year in lost productivity and health care expenditures (Centers for Disease Control and Prevention [CDC] 2002, 2005). Multiple factors contribute to the initiation and maintenance of cigarette smoking (Li 2006). One personality factor that is consistently associated with vulnerability to and severity of smoking is impulsivity (e.g., Flory and Manuck 2009; Mitchell 1999; Nieva et al. 2011). Impulsivity is a multi-faceted concept that includes inability to wait, difficulty in refraining from actions, and insensitivity to delayed consequences (de Wit 2008).

Insensitivity to delayed consequences is encompassed by the process of *delay discounting*: the decline in the present value of a reward with delay to its receipt (e.g., Mazur 1987; Odum 2011a). In humans, delay discounting is often investigated by asking the participant to choose between a smaller, more immediate alternative and a larger, more delayed alternative. Across choice opportunities, the experimenter changes the amount of the immediate option until an indifference point is reached. At indifference, the amount of the smaller-sooner outcome provides the present value of the larger-later outcome. If delayed outcomes hold comparatively little value, a person would choose smaller more immediate outcomes relatively often, which is deemed impulsive choice (e.g., Logue 1988).

Cigarette smoking is strongly related to delay discounting. For example, current smokers more steeply discount delayed money than do non-smokers (e.g., Baker et al. 2003; Bickel et al. 1999; Heyman and Gibb 2006; Mitchell 1999; Ohmura et al. 2005; Reynolds and Fields 2012; Wing et al. 2012; see Mackillop et al. 2011 for review and meta-analysis). Furthermore, the degree of discounting of delayed money is predictive of smoking initiation and likelihood of success in quitting. For example, in a prospective longitudinal study, adolescents who steeply discounted delayed money were more likely to begin smoking by young adulthood than adolescents who discounted money less steeply (Audrain-McGovern et al. 2009). In a laboratory analog model of relapse to smoking, in which participants are paid for remaining abstinent, steeper discounting of delayed money is predictive of shorter latency to smoke (Dallery and Raiff 2007; Mueller et al. 2009). Additionally, steep discounting of delayed money is predictive of poorer treatment outcome for cigarette dependence in clinical settings (MacKillop and Kahler 2009; Sheffer et al. 2012; Yoon et al. 2007). Thus, steep delay discounting is associated with cigarette smoking and predicts important outcomes for cigarette smokers. With better understanding, delay discounting could provide a vital role in the development of prevention and cessation strategies.

Although steep discounting of delayed hypothetical money is a robust feature associated with cigarette smoking, in some ways the generality of this relation has been little investigated. The difference between smokers and nonsmokers in discounting of money, for example, could reflect smokers’ intent to purchase cigarettes with at least a portion of the money. Several studies have revealed that smokers discount cigarettes very steeply (e.g., Bickel et al. 1999; Odum and Baumann 2007) and if money and cigarettes are treated as partially equivalent, then steep discounting of delayed money may reflect no more than this tendency to steeply discount delayed cigarettes.

In the present study, we evaluated if, relative to nonsmokers, cigarette smokers more steeply discount a variety of delayed outcomes. Specifically, in addition to delayed money, we compared how current cigarette smokers and non-smokers discounted delayed alcohol, food, and entertainment. These commodities were chosen because they are widely available and consumed, but are unlikely to be exchanged directly for cigarettes. On one hand, if cigarette smokers discount non-monetary outcomes more steeply than do non-smokers, then this result could support the hypothesis that delay discounting is a pervasive trait-like tendency (see Odum 2011a, b). On the other hand, if cigarette smokers discount only money more steeply than do non-smokers, then this result could suggest that smokers may show steep discounting of money in part because it is used to purchase cigarettes.

## Method

### Participants

A total of 65 participants (32 smokers, 33 non-smokers) were recruited through a combination of newspaper advertisements, radio advertisements, fliers posted throughout the community, and referrals from other participants. By telephone, potential participants were asked a series of questions to determine if they qualified.

Only occasional alcohol drinkers 21 years or older were invited to come to the laboratory to participate. Non-smokers reported having smoked fewer than 100 cigarettes in their lifetime and smokers reported smoking at least 10 cigarettes/day (CDC 2006). People who met these qualifications were invited to the laboratory for additional screening and testing.

### Procedure

Each participant completed a single session while seated at a desk with a computer in a private office with no windows. Participants read and signed an informed consent form that was approved by Utah State University’s Institutional Review Board. Participants were compensated $25 for completing the approximately 1-h session.

#### Biological samples

Participants first provided two biological samples. The first sample, administered through the FC 10 Breathalyzer (Lifeloc), measured recent alcohol consumption. Any participant with a blood-alcohol level above 0.000 was not included in the study (one participant was excluded based on this criterion). The second sample, administered with a Micro+ Smokerlyzer (Bedfont Scientific LTD.), measured carbon monoxide (CO) as an indication of recent cigarette use. Reported smokers had to measure a CO level of 6 ppm or higher (Bedfont Scientific n.d.) to qualify for participation. All smokers met this criterion.

#### Questionnaires

Next, participants completed a series of questionnaires on the computer. The questionnaires were administered through E-Prime computing software.

The Eating Disturbance Scale (EDS-5; Rosenvinge et al. 2001) is a five-item questionnaire that measures problematic eating habits and beliefs (*α* = 0.666). Questions include: “Are you satisfied with your eating habits?” Scores can range from 5 to 35.

The South Oaks Gambling Screen (SOGS; Lesieur and Blume 1987) is a 36-item questionnaire that measures gambling behavior (with an answer scale of “not at all,” “less than once a week,” and “once a week or more”; *α* = 0.812). Questions include: “In your lifetime, how often have you gone to a casino (legal or otherwise)?” Scores can range from 0 to 20.

The Michigan Alcohol Screening Test (MAST; Selzer 1971) is a 25-item questionnaire that identifies alcohol abuse in respondents using “yes” or “no” questions (*α* = 0.888). All questions are based on the participants’ experience in their lifetime. Questions include “Has your significant other (or other family member) gone to anyone for help about your drinking?” Scores can range from a minimum of 0 to a maximum of 53 (answering yes to specific questions is weighted more than other questions).

The Information Inventory (II; Altus 1948) is a 13-item IQ questionnaire that asks a variety of questions ranging from events in history to vocabulary. Sample questions include “Who was Confucius?” Scores can range from 0 to 30.

Participants also provided demographic information including their age, ethnicity, gender, marital status, income, and highest obtained education.

#### Delay discounting tasks

In the final portion of the session, participants completed four different delay discounting tasks on the computer. Prior to the delay discounting tasks, the participants read instructions similar to those in described in Odum et al. (2006). The tasks were presented in randomly determined order and all four were completed in approximately 40 min. Before the first discounting task, participants completed a ten-question practice block with money. The delay to the larger-later reward of $100 was set to 1 week and the immediate amount increased from $10 to $100 in $10 increments across practice trials.

In each delay discounting task indifference points were obtained at six different delays to the larger-later reward, presented in the following order: 1 week, 2 weeks, 1 month, 6 months, 5 years, and 25 years. For the monetary task the first question was, “Would you prefer $50 now or $100 in (delay)?” The positioning of the immediate and delayed options alternated randomly across the right and left positions of the computer screen. Participants registered their choice by using the mouse to click one of the two options. After each question, the amount of the immediate reward was adjusted according to the titration procedure outlined by Du et al. (2002). Briefly, if the smaller-sooner reward was selected (forgone), the amount of that reward was decreased (increased) by $25 in the next choice trial. Subsequent adjustments to the immediate reward were 50 % of the preceding adjustment. The amount of the immediate reward following the tenth choice trial was used as the indifference point for that delay. At each subsequent delay, the amounts of the smaller-sooner and larger-later rewards were returned to $50 and $100, respectively, and the ten-trial titration procedure was repeated. All values displayed to participants were rounded to the nearest penny ($0.01).

The other three delay discounting tasks asked about different commodities: food, alcohol, or entertainment. For each task, the participant was asked to name their favorite item in the commodity category (e.g., favorite alcoholic drink) and to report how much that item cost. The reported cost was then divided into $100, and the quotient served as the larger-later reward amount throughout that discounting task, similar to the procedure first used by Odum and Rainaud (2003). The initial amount of the smaller-sooner reward was half the amount of the larger-later. For example, if a participant indicated that their favorite food was a hamburger and that it cost $5, their first question would read “Would you rather have 10 servings of hamburger now or 20 servings of hamburger in one week?” From there, the titration procedure outlined above was used to obtain indifference points for that commodity at each delay. Across outcomes all indifference points were scaled by the amount of the larger, later outcome so that all indifference points reported are standardized between 0 and 1. Favorite foods reported by participants included bread, enchiladas, and fish. Participants’ reports of favorite alcohol included beer, long island iced tea, and wine. Favorite entertainment reported by participants included mp3’s, iTunes albums, and CDs.

No major changes were made to the Du et al. (2002) discounting task across commodities. Each indifference point was determined by a ten-trial titration procedure and all values were rounded to the nearest hundredth. If a participant chose a relatively expensive favorite commodity, that commodity would have a small number of items that could be purchased with $100. Therefore, as an unintended consequence, a participant could be given a choice in which the smaller, sooner option did not change across a trial because the titration amount was less than 0.01. Of 1,560 indifference points (i.e., four commodities tested at six delays for 65 participants), a total of 33 indifference points were affected by this issue. In these cases, the titration procedure would have effectively stopped at trial 9, for example, rather than trial 10.

### Analyses

The Mazur (1987) hyperbola and Myerson and Green (1995) hyperboloid model were fit to the median indifference points for each commodity via curvilinear regression (Graphpad Prism®):

1 $$ V=\frac{A}{{\left(1+ kD\right)}^s} $$

where *V* is the present (discounted) value of a future outcome, *A* is the amount of that future outcome, *D* is the delay to that outcome, *k* quantifies steepness of the hyperboloid delay discounting function and *s* is a scalar of delay and/or amount. The key difference between the two models is that the Mazur (1987) hyperbola has no exponential scaling parameter (so *s* was constrained to 1 for the model fit). To select the appropriate model for analyses, the models were compared with the Akaike Information Criterion (AIC; described in the “Results” section).

For reasons described below, a general linear model (GLM) was used as a repeated-measures analysis of variance (ANOVA) to examine the effects of smoking status and type of outcome (e.g., money) on the indifference points (cf. Evenden and Ryan 1996). For the omnibus test of smoking status, all the indifference points obtained from non-smokers (24 for each participant) were averaged and compared to all of the average indifference points obtained from smokers. For *between-group* pairwise comparisons, all indifference points (6 for each participant) for one outcome type (e.g., money) for non-smokers were averaged and compared to all of the average indifference points obtained for that outcome type for smokers, resulting in four between-group pairwise comparisons. For *within-group* pairwise comparisons, for each group (e.g., non-smokers) average indifference points for each outcome (e.g., money) were compared to the average indifference points for the other outcomes (e.g., food), resulting in six within-group comparisons for each group. In the “Results” section, all comparisons are reported as the difference between the means under consideration (e.g., mean indifference points for non-smokers minus mean indifference points for smokers).

GLM was chosen due to its ability to analyze repeated measures and provide pairwise comparisons while adjusting for multiple comparisons. The family-wise Type I error rate was held constant at *p* = 0.05. AUC was not used in a more standard ANOVA for this analysis because (a) Shapiro–Wilk tests revealed that AUC for all commodities violated the assumption of normality (*p* < 0.01), which is an assumption of ANOVA and (b) there is no widely available or accepted non-parametric omnibus test that accounts for multiple comparisons and provides pairwise comparisons (a minimum of two Friedman’s tests and seven Mann–Whitney *U*-tests would be required to non-parametrically provide the information reported by the GLM). The profile of results with AUC was the same as presented here with indifference points. We were not able to use the value of the free-parameter *k* from Eq. 1 for these analyses for two reasons. (1) As described below, the Myerson and Green (1995) hyperboloid was determined to be the best model and (2) in the hyperboloid model the value of *k* interacts with the value of *s*, so *k* does not provide an independent measure of the degree discounting.

To be consistent with prior studies examining within-subject relations between the discounting of different commodities (Charlton and Fantino 2008; Johnson et al. 2010; Odum 2011b), correlation coefficients were computed using the area under the curve (AUC) as the measure of delay discounting. AUC is the sum of the trapezoidal area between each set of adjacent indifference points. The formula for a single trapezoid is *x*_2_ – *x*_1_[(*y*_1_ + *y*_2_)/2], where *x*_1_ and *x*_2_ are successive delays and *y*_1_ and *y*_2_ are indifference points associated with those delays. AUC is standardized to fall between 0 and 1, with lower values indicating steeper delay discounting (Myerson et al. 2001). Within-subject correlations between discounting of different commodities could not be computed with parameters from the Myerson and Green (1995) model. The parameters *k* and *s* from this model (Eq. 1) interact and neither parameter provides an independent measure of the degree of discounting.

We did not exclude any of the discounting data obtained from participants from analysis for two reasons. First, the present study is an extension of Bickel et al. (1999), which predated the data exclusion criteria developed by Johnson and Bickel (2008). Second, due to the within-subjects nature of many comparisons in the present study, if a participant had data that met exclusion criteria for one outcome type, all four of that person’s discounting curves (one for each outcome type) would have to be excluded. This strategy would necessarily exclude a large amount of systematic data. The pattern of results was the same regardless whether we included or excluded data according to the Johnson and Bickel algorithm. Thus, for these reasons, we did not exclude any data.

## Results

Demographic characteristics and mean questionnaire scores of the smoker and non-smoker groups are shown in Table 1. Fifty-two participants self-identified as Caucasian (80 %), four reported as Hispanic and one as African American and six as “other.” Additionally, three participants self-identified as Latino. Reported ethnicity did not differ between groups (*χ* ^2^ (3, *N* = 63) = 2.73, *p* = 0.44). The groups differed with respect to MAST and SOGS scores, with smokers reporting greater problematic alcohol use and gambling. Therefore, MAST and SOGS scores were included in the GLM as covariates. A chi-square test for independence did not reveal gender differences between the non-smoker (18 males, 14 females) and smoker (19 males, 12 females) groups, *χ* ^2^ (1, *N* = 63) = 0.17, *p* = 0.69. For biological samples, smokers had higher CO levels than non-smokers [*t*(61) = 20.39, *p* < 0.01], but did not differ in BAL, which was required to be 0.000 for participation.

**Table 1:** Means and standard errors for demographics, questionnaire results, and CO levels, separated by group

Means and standard errors of questionnaires			
	Non-smoker mean (SE)	Smoker mean (SE)	*t*
Caucasian	84 %	81 %	
Male	56 %	61 %	
Education^a^	3.13 (0.22)	2.65 (0.17)	1.68+
Age (years)	38.38 (2.79)	36.90 (2.51)	0.39
Monthly income ($)	2,080 (301)	2,055 (447)	.047
Information Inventory	10.13 (0.58)	8.52 (0.63)	1.88
MAST^b^	3.00 (0–8)	12 (0–33)	197.00*
SOGS^b^	0.00 (0–1.75)	1.00 (0–5)	292.50*
CO (ppm)	1.97 (0.13)	9.35 (0.34)	−20.15^c^*

^a^Education was measured using seven categories and participants were asked about their highest level of obtained education: 1 = did not complete high school, 2 = high school degree or equivalent, 3 = associate degree, 4 = bachelors degree, 5 = graduate degree, 6 = doctorate degree or equivalent

^b^Median and interquartile ranges (25 % and 75 % percentiles) reported instead of mean and standard error. Shapiro–Wilk test for normality indicates that scores are not normally distributed. Non-parametric Mann–Whitney *U*-test reported in place of *t*-test

^c^Violation of Levene’s Test for Equality of Variances, equal variances not assumed

*p < 0.05

Table 2 allows evaluation of the fit of two common models of delay discounting to the indifference points. The left column shows the value of the AIC for the fit of the Mazur (1987) hyperbolic model and hyperboloid Myerson and Green (1995) model to the median indifference points. The AIC weighs how much variance is accounted for in light of how many free parameters a model has. AIC values from the hyperboloid model were less than AIC values from the hyperbolic model, indicating a superior fit, for five out of eight comparisons. For model fits to the individual participant data, the right column of Table 2 shows the median *R* ^2^ values for the hyperboloid model were exclusively higher than for the hyperbolic model. AIC was also calculated for model fits to indifference points for each participant and commodity. AIC values indicated a superior fit for the Myerson and Green (1995) model 182 (70 %) out of 260 individual data sets. We used a binomial test to determine the likelihood of obtaining this distribution of AIC scores by chance. For the binomial test we assumed that each model was equally likely to be the best fit to individual participant data (*p* = 0.5). The results of the binomial test indicate that our distribution of results are likely not due to chance (*p* < 0.001). For these reasons, the Myerson and Green (1995) hyperboloid model was selected for analyses.

**Table 2:** Model fit comparisons for the Mazur (1987) hyperbola and Myerson and Green (1995) hyperboloid (see text)

		AIC		Median R^2^	
	Outcome	Mazur (1987)	Myerson and Green (1995)	Mazur (1987)	Myerson and Green (1995)
Non-smoker	Money	9.11	**7.57**	0.96	**0.98**
	Alcohol	**14.92**	15.97	0.52	**0.75**
	Entertainment	**5.69**	7.68	0.87	**0.95**
	Food	15.90	**13.68**	0.63	**0.80**
Smoker	Money	15.74	**14.32**	0.74	**0.82**
	Alcohol	17.63	**16.43**	0.12	**0.54**
	Entertainment	**12.08**	12.99	0.70	**0.85**
	Food	8.31	**6.94**	0.38	**0.61**

Values presented in *bold* indicate the better fit. For median indifference points, the Akaike information criterion (AIC) results indicate that the hyperboloid provided a better fit five out of eight times. Comparisons of R^2^ values obtained from fitting both models to individual participant data indicate that the hyperboloid fit better in all cases than the hyperbola

**Fig. 1:**
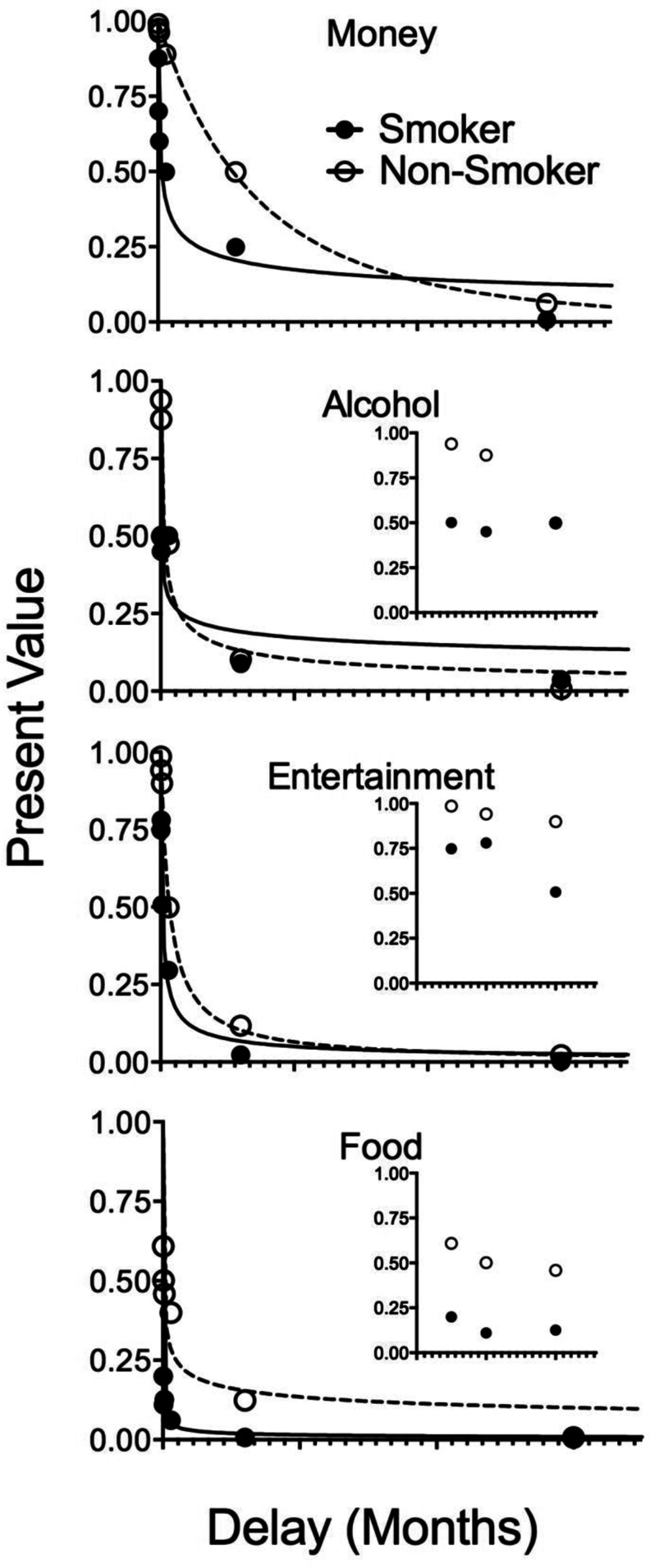
Discounting functions for smokers and non-smokers for the commodities of money, alcohol, food, and entertainment. In all four panels, the points show median indifference points and lines show the best fitting hyperbola like discounting function (Myerson and Green 1995). Insets for the commodities of alcohol, entertainment, and food are the same data with the *x*-axis scaled to show indifference points at the shortest delays. In some cases, data points may overlap

Figure 1 shows the median indifference points, expressed as a proportion of the delayed reward amount at each delay, in the four delay discounting tasks for smokers and non-smokers. The insets in the panels for alcohol, entertainment, and food constrain the *x*-axis to more clearly show the indifference points at the shortest delays. The hyperboloid decay functions (Myerson and Green 1995) were fit to the median indifference points. Table 3 lists the obtained best-fit parameters of Eq. 1, *k* and *s* for each group and commodity as well as goodness of fit of the model, *R* ^2^. The hyperboloid model had *R* ^2^ values that were greater than 0.9 for seven of the eight data sets obtained, with the model performing relatively poorly for alcohol for smokers (*R* ^2^ = 0.71).

**Table 3:** The *k* and *s* parameters as well as *R* ^*2*^ for hyperboloid model (Myerson and Green 1995) fits to median indifference points for each outcome for each group

	Outcome	*k*	*s*	*R* ^*2*^
Non-smoker	Money	0.004	3.61	0.99
	Alcohol	1.30	0.47	0.92
	Entertainment	0.16	0.97	0.99
	Food	19.91	0.26	0.91
Smoker	Money	3.28	0.30	0.94
	Alcohol	66.18	0.20	0.71
	Entertainment	1.71	0.58	0.97
	Food	206.30	0.42	0.94

Between-group differences in indifference points are considered next for the GLM analysis. Across outcomes, smokers discounted delayed outcomes more than did non-smokers (significant main effect of group, *F*(2.57, 149.22) = 8.52, *p* < 0.01, *ηρ* ^2^ = 0.13), with standardized indifference points for non-smokers averaging 0.14 greater than that of smokers. Pairwise comparisons revealed that nonsmokers’ indifference points were significantly greater than those of smokers on the discounting tasks involving money (mean difference [MD] = 0.22, *p* < 0.01) food (MD = 0.17, *p* < 0.05) and entertainment (MD = 0.16, *p* < 0.05) but not alcohol (MD = −0.03, *p* = 0.73). Mauchly’s Test of Sphericity was statistically significant for two out of the three comparisons, indicating that the variance in the indifference points for at least one of the commodities was significantly different from that of the other commodities. Therefore, the more conservative Greenhouse–Geisser *F* test is reported. The use of the Greenhouse–Geisser *F* tests did not alter the results of the GLM.

To investigate differences in discounting of the different commodities within groups, pairwise comparisons were analyzed using mean indifference points for each outcome (Table 4). For non-smokers, indifference points obtained for money were greater than indifference points obtained for alcohol, entertainment, and food. For smokers, the indifference points for money were greater than the indifference points for the food, but the indifference points for money were not different from those for alcohol or entertainment. Thus, when comparing money to other outcomes for non-smokers, all of the commodities were significantly different than money, whereas for smokers, only food was significantly different than money.

**Table 4:** Mean difference of indifference points obtained from the general linear model (GLM) organized by group

Group	Mean difference			
		Alcohol	Entertainment	Food
Non-smoker	Money	0.28***	0.13*	0.27***
	Food	0.05	−0.15*	
	Entertainment	0.15*		
Smoker	Money	0.03	0.07	0.20*
	Food	−0.18	−0.14	
	Entertainment	−0.04		

*p < 0.05; ***p < 0.001

To examine whether a person’s discounting of one outcome was related to discounting of another outcome, within group correlations between AUC values obtained with the different commodities were conducted (Table 5). The Spearman rho correlation was used because AUC was not normally distributed. All within-group correlations between commodities were statistically significant. For non-smokers, the effect sizes for all of the correlations are in the medium range (*r* = between 0.3 and 0.5). For smokers, three of the effect sizes are medium while the other three are large (*r* > 0.5). Therefore, within individuals, delay discounting for one commodity was associated with discounting of other commodities. That is, a person who tended to discount one outcome steeply also tended to show steep discounting for other outcomes, and a person who tended to discount one outcome shallowly also tended to show shallow discounting for other outcomes.

**Table 5:** Spearman correlations of area under the curve by group

Group	Spearman correlations			
		Alcohol	Entertainment	Food
Non-smoker	Money	0.34*	0.41*	0.37*
	Food	0.35*	0.42**	
	Entertainment	0.37*		
Smoker	Money	0.42*	0.65**	0.50**
	Food	0.38*	0.68**	
	Entertainment	0.38*		

For both groups, AUC for one commodity was predictive of other AUC values within that group

*p ≤ 0.05; **p < 0.01

## Discussion

In this study, we compared for the first time how cigarette smokers and non-smokers discounted tangible outcomes (food, entertainment, and alcohol). We found that smokers discounted two of these commodities (food and entertainment) more steeply than non-smokers did, showing that the tendency for steep discounting by cigarette smokers extends across domains. We also replicated previous findings that smokers discount money more steeply than non-smokers do, that people discount money less steeply than other outcomes, and that a person’s degree of discounting one delayed outcome is related to that person’s degree of discounting other outcomes. Below, we discuss each of these findings in turn.

Our results replicate and extend the result that cigarette smokers discount money more steeply than non-smokers do (e.g., Bickel et al. 1999; Mitchell 1999). Steeper discounting of money by smokers has been found across a variety of populations, amounts of money, delays, and procedural methods (see MacKillop et al. 2011). Furthermore, cigarette smokers discount health outcomes (Baker et al. 2003; Odum et al. 2002), as well as money for a group of people including themselves, more steeply than do non-smokers (Bickel et al. 2012a). Despite the generality of these effects, prior research has shed little light on the source of these differences. We elucidated the finding that smokers discount money more steeply than non-smokers do by extending it to food and entertainment. Smokers discount money more steeply than non-smokers, but not necessarily because money is a means to purchase cigarettes. Instead, smokers may show heightened discounting of multiple types of delayed outcomes.

In the current experiment, cigarette smokers also discounted alcohol nominally (but not significantly) more steeply than non-smokers did. There are several possible reasons for this finding. Perhaps a difference exists between their discounting of this commodity, but our measure of delay discounting was not sensitive enough to detect it. Although this explanation is plausible, we were able to detect differences in discounting with our measures with the other commodities. One possible procedural modification that could help differentiate steeply discounted commodities would be to include shorter delays to the larger-later reward. This inclusion would allow more fine-grained distinctions between discounting over shorter time frames.

Another possibility is that for some as yet unknown reason, smokers and non-smokers do not differ in the degree to which they discount alcohol, but they do differ in the degree to which they discount other things. This finding seems unlikely, but possible, given the pattern of discounting for other things, and also given the nominal but non-significant differences in discounting for alcohol. Further research is needed to determine the nature and degree of differences, if any, in delay discounting for alcohol by smokers and non-smokers.

Our results add to the growing number of findings that steeply discounting one delayed commodity is predictive of how steeply other commodities will be discounted (Charlton and Fantino 2008; Johnson et al. 2010; Odum 2011b). For example, in a comprehensive analysis of data from prior studies, Odum (2011b) found that colleges students who tended to discount money steeply, also tended to discount food steeply. Similarly, opioid-dependent outpatients who showed steep discounting of money also discounted heroin steeply. Community members who discounted money more steeply similarly discounted alcohol, and discounting of money and food was related to discounting of alcohol. Finally, cigarette smokers who showed steep discounting of money also showed steep discounting of cigarettes. The results of the present study replicate and extend those from our laboratory and others showing that a person who shows precipitous loss of value with delay in one domain will also likely show similar changes in value with delay to an outcome in another domain.

Together, these results extend support for our suggestion that in addition to showing strong state (environmental) influences, delay discounting may also have a trait-like component (Odum 2011a, b). A state influence is an environmental manipulation that affects behavior over a relatively short time frame (see, e.g., Odum and Baumann 2010). There are robust state influences on delay discounting, including the amount of an outcome, whether it is gained or lost, and the context in which the choice is made. The present study provides a clear example of state influences on discounting in the differences between discounting for money and other outcomes. The same people, in a relatively short time frame, can show steep discounting for food, for example, and then more moderate discounting by delay with money (see also Estle et al. 2007; Odum and Rainaud 2003; Odum et al. 2006).

Delay discounting also shows clear trait influences. A trait may be defined as “a relatively enduring pattern of thoughts, feelings, and behaviors that reflects the tendency to respond in certain ways under certain circumstances” (Roberts 2009). To address the first part of the definition of a trait, delay discounting is relatively enduring in the sense that it is by and large stable across the time frames in which it has thus far been measured (e.g., up to 1 year as in Kirby 2009; see Odum 2011b for discussion). The present study and others showing strong correlations in the degree of discounting for one type of outcome and degree of discounting for another type of outcome constitute evidence for the second part of the definition of a trait, that it reflects the tendency to respond certain ways under certain circumstances.

Our interpretation of these results is consistent with the view that steep discounting is trans-disease process (Bickel et al. 2012b). The view that discounting is a trans-disease process points to patterns of steep discounting *across* many psychological disorders. The view that discounting is like a trait points to discounting patterns *within* a person that extend through time and across the outcome being discounted. A person who tends to prefer immediate but reduced rewards in one area will also tend to choose immediate but reduced rewards in another area, and have a higher risk of psychopathology. The converse is also true, that a person who prefers to wait for a larger reward in the future in one domain will also prefer to wait for a larger reward in the future in other domains, and will have a lower risk of psychopathology.

One possible limitation of the present experiment is the amount of the rewards that we used in the delay discounting assessments. For example, most people may rarely consume $100 worth of food or alcohol at one time. While the choices in the consumable-commodity discounting tasks may have seemed less plausible than those in the monetary discounting task, Odum et al. (2006) showed that people discounted $10 worth of food more steeply than money. Because we replicated this difference between discounting of larger amounts of food and money in both the smoker and nonsmoker groups, it appears that the implausibility of consuming large amounts of food, for example, does not compromise our findings.

In general, cigarette smokers tend to consume more alcohol than do non-smokers (e.g., DiFranza and Guerrera 1990; Carmody et al. 1985). In the present study, smokers had higher MAST scores (indicating that they had greater and more problematic alcohol use) than non-smokers did. Few prior studies comparing delay discounting as a function of smoking status have reported alcohol consumption, so there is little basis in the literature to evaluate the contribution of this difference to the present results. We included MAST scores as a covariate in our analyses of indifference points, thus providing statistical control of its influence in our results. Furthermore, the results of the analyses were the same when we included MAST scores as a covariate and when we did not. Thus, it is currently unclear how concomitant alcohol use contributes to steep discounting in smokers.

In the present study, cigarette smokers also had higher SOGS scores, indicating more gambling activity and problems associated with gambling, than non-smokers. This finding is consistent with prior results showing that pathological gamblers discount hypothetical money more steeply, and smoke more heavily, than non-gamblers (e.g., Petry 2001; Rodda et al. 2004). Future studies could include participants with a wider range of SOGS scores to address the interaction of gambling and smoking status in determining the degree of discounting of different outcomes.

In conclusion, we found that relative to non-smokers, cigarette smokers more steeply discounted delayed money, food, and entertainment. This finding is important in clarifying prior findings of more impulsive decision making for delayed money by smokers compared to non-smokers. One possibility was that because smokers can spend a substantial portion of their income on cigarettes (e.g., Steinberg et al. 2004), steeper discounting of money merely reflected the use of money to purchase cigarettes. This hypothesis was not supported. Instead, cigarette smokers also discount other outcomes more steeply than non-smokers do, suggesting that smokers may show relatively pervasive steep discounting of delayed outcomes in general.

How much a person discounts an outcome when it is delayed is a potentially powerful measure. Degree of delay discounting is associated with a variety of social maladies, including drug addiction, obesity, problematic gambling, as well as reduced academic performance, self care, and personal safety (see Bickel et al. 2012a; Odum 2011b, for a summary). Furthermore, steepness of delay discounting is predictive of a person’s likelihood of initiating as well as overcoming substance abuse (e.g., Audrain-McGovern et al. 2009; MacKillop and Kahler 2009). Degree of delay discounting appears to be heritable (e.g., Anokhin et al. 2011; Madden et al. 2008; Wilhelm and Mitchell 2009), and thus have a genetic component. Delay discounting may also be modifiable by a variety of techniques (e.g., Bickel et al. 2011; Black and Rosen 2011; Koffarnus et al. 2013; Morrison et al. 2014; Stein et al. 2013). Thus, how much a person values an outcome when delayed could serve as an important diagnostic as well as outcome measure.

## References

[CR1] Altus WD (1948). The validity of an abbreviated information test used in the Army. J Consult Psychol.

[CR2] Anokhin AP, Golosheykin S, Grant JD, Heath AC (2011). Heritability of delay discounting in adolescence: a longitudinal twin study. Behav Genet.

[CR3] Audrain-McGovern J, Rodriguez D, Epstein LH (2009). Does delay discounting play an etiological role in smoking or is it a consequence of smoking?. Drug Alcohol Depend.

[CR4] Baker F, Johnson MW, Bickel WK (2003). Delay discounting in current and never-before cigarette smokers: similarities and differences across commodity, sign, and magnitude. J Abnorm Psychol.

[CR5] Bedfont Scientific LTD. (n.d.) Micro+ smokerlyzer operating manual. Retrieved from www.bedfont.com

[CR6] Bickel WK, Odum AL, Madden GJ (1999). Impulsivity and cigarette smoking: delay discounting in current, never, and ex-smokers. Psychopharmacology.

[CR7] Bickel WK, Yi R, Landes RD (2011). Remember the future: working memory training decreases delay discounting among stimulant addicts. Biol Psychiatry.

[CR8] Bickel WK, Jarmolowicz DP, Mueller ET (2012). Are executive function and impulsivity antipodes? A conceptual reconstruction with special reference to addiction. Psychopharmacology.

[CR9] Bickel WK, Jarmolowicz DP, Mueller ET (2012). Excessive discounting of delayed reinforcers as a trans-disease process contributing to addiction and other disease-related vulnerabilities: emerging evidence. Pharmacol Ther.

[CR10] Black AC, Rosen MI (2011). A money management-based substance use treatment increases valuation of future rewards. Addict Behav.

[CR11] Carmody TP, Brischetto CS, Matarazzo JD (1985). Co-occurrent use of cigarettes, alcohol, and coffee in healthy, community-living men and women. Health Psychol.

[CR12] Centers for Disease Control and Prevention (2002) Annual smoking-attributable mortality, years of potential life lost, and economic costs—United States, 1995–1999. In: Morb Mortal Wkly Rep: 51. Available via http://www.cdc.gov/mmwr/preview/mmwrhtml/mm5114a2.htm. Accessed 16 Sept 2013 12002168

[CR13] Centers for Disease Control and Prevention (2005) Annual smoking-attributable mortality, years of potential life lost, and productivity losses—United States, 1997–2001. Morb Mortal Wkly Rep: 54. Available via http://www.cdc.gov/mmwr/preview/mmwrhtml/mm5425a1.htm. Accessed 16 Sept 2013

[CR14] Centers for Disease Control and Prevention (2006). Tobacco use among adults—United States, 2005. Morb Mortal Wkly Rep.

[CR15] Charlton SR, Fantino E (2008). Commodity specific rates of temporal discounting: does metabolic function underlie differences in rates of discounting?. Behav Process.

[CR16] Dallery J, Raiff BR (2007). Delay discounting predicts cigarette smoking in a laboratory model of abstinence reinforcement. Psychopharmacology.

[CR17] de Wit H (2008). Impulsivity as a determinant and consequence of drug use: a review of underlying processes. Addict Biol.

[CR18] DiFranza JR, Guerrera MP (1990). Alcoholism and smoking. J Stud Alcohol Drugs.

[CR19] Du W, Green L, Myerson J (2002). Cross-cultural comparisons of discounting delayed and probabilistic rewards. Psychol Rec.

[CR20] Estle SJ, Green L, Myerson J, Holt DD (2007) Discounting of monetary and directly consumable rewards. Psychol Sci 18:58–63 10.1111/j.1467-9280.2007.01849.x 17362379

[CR21] Evenden JL, Ryan CN (1996). The pharmacology of impulsive behaviour in rats: the effects of drugs on response choice with varying delays of reinforcement. Psychopharmacology.

[CR22] Flory JD, Manuck SB (2009). Impulsiveness and cigarette smoking. Psychosom Med.

[CR23] Heyman GM, Gibb SP (2006). Delay discounting in college cigarette chippers. Behav Pharmacol.

[CR24] Johnson MW, Bickel WK (2008). An algorithm for identifying nonsystematic delay-discounting data. Exp Clin Psychopharmacol.

[CR25] Johnson MW, Bickel WK, Baker F (2010). Delay discounting in current and former marijuana-dependent individuals. Exp Clin Psychopharmacol.

[CR26] Kirby KN (2009). One-year temporal stability of delay-discount rates. Psychon Bull Rev.

[CR27] Koffarnus MN, Jarmolowicz DP, Mueller ET (2013). Changing delay discounting in the light of the competing neurobehavioral decision systems theory: a review. J Exp Anal Behav.

[CR28] Lesieur HR, Blume SB (1987). The South Oaks Gambling Screen (SOGS): a new instrument for the identification of pathological gamblers. Am J Psychiatr Rehabil.

[CR29] Li MD (2006). The genetics of nicotine dependence. Curr Psycyhiatry Rep.

[CR30] Logue AW (1988). Research on self-control: an integrating framework. Behav Brain Sci.

[CR31] MacKillop J, Kahler CW (2009). Delayed reward discounting predicts treatment response for heavy drinkers receiving smoking cessation treatment. Drug Alcohol Depend.

[CR32] MacKillop J, Amlung MT, Few LR (2011). Delayed reward discounting and addictive behavior: a meta-analysis. Psychopharmacology.

[CR33] Madden GJ, Smith NG, Brewer AT (2008). Steady-state assessment of impulsive choice in Lewis and Fischer 344 rats: between-condition delay manipulations. J Exp Anal Behav.

[CR34] Mazur JE, Commons ML, Mazur JE, Nevin JA, Rachlin H (1987). An adjusting procedure for studying delayed reinforcement. Quantitative analysis of behavior.

[CR35] Mitchell SH (1999). Measures of impulsivity in cigarette smokers and non-smokers. Psychopharmacology.

[CR36] Morrison KL, Madden GJ, Odum AL (2014). Altering impulsive decision making with an acceptance-based procedure. Behav Ther.

[CR37] Mueller ET, Landes RD, Kowal BP (2009). Delay of smoking gratification as a laboratory model of relapse: effects of incentives for not smoking, and relationship with measures of executive function. Behav Pharmacol.

[CR38] Myerson J, Green L (1995). Discounting of delayed rewards: models of individual choice. J Exp Anal Behav.

[CR39] Myerson J, Green L, Warusawitharana M (2001). Area under the curve as a measure of discounting. J Exp Anal Behav.

[CR40] Nieva G, Valero S, Bruguera E (2011). The alternative five-factor model of personality, nicotine dependence and relapse after treatment for smoking cessation. Addict Behav.

[CR41] Odum AL (2011). Delay discounting: I’m a k, you’re a k. J Exp Anal Behav.

[CR42] Odum AL (2011). Delay discounting: trait variable?. Behav Process.

[CR43] Odum AL, Baumann AAL (2007). Cigarette smokers show steeper discounting of both food and cigarettes than money. Drug Alcohol Depend.

[CR44] Odum AL, Baumann AAL, Madden GJ, Bickel WG (2010). Delay discounting: state and trait variable. Impulsivity: the behavioral and neurological science of discounting.

[CR45] Odum AL, Rainaud CP (2003). Discounting of delayed hypothetical money, alcohol, and food. Behav Process.

[CR46] Odum AL, Madden GJ, Bickel WK (2002). Discounting of delayed health gains and losses by current, never- and ex-smokers of cigarettes. Nicotine Tob Res.

[CR47] Odum AL, Baumann AAL, Rimington DD (2006). Discounting of delayed hypothetical money and food: effects of amount. Behav Process.

[CR48] Ohmura Y, Takahashi T, Kitamura N (2005). Discounting delayed and probabilistic monetary gains and losses by smokers of cigarettes. Psychopharmacology.

[CR49] Petry NM (2001). Pathological gamblers, with and without substance use disorders, discount delayed rewards at high rates. J Abnorm Psychol.

[CR50] Reynolds B, Fields S (2012). Delay discounting by adolescents experimenting with cigarette smoking. Addiction.

[CR51] Roberts BW (2009). Back to the future: personality and assessment and personality development. J Res Pers.

[CR52] Rodda S, Brown SL, Phillips JG (2004). The relationship between anxiety, smoking, and gambling in electronic gaming machine players. J Gambl Stud.

[CR53] Rosenvinge JH, Perry JA, Bjørgum L (2001). A new instrument measuring disturbed eating patterns in community populations: development and initial validation of a five‐item scale (EDS‐5). Eur Eat Disord Rev.

[CR54] Selzer ML (1971). The Michigan alcoholism screening test: the quest for a new diagnostic instrument. Am J Psychiatry.

[CR55] Sheffer C, MacKillop J, McGeary J (2012). Delay discounting, locus of control, and cognitive impulsiveness independently predict tobacco dependence treatment outcomes in a highly dependent, lower socioeconomic group of smokers. Am J Addict.

[CR56] Stein JS, Johnson PS, Renda CR (2013). Early and prolonged exposure to reward delay: effects on impulsive choice and alcohol self-administration in male rats. Exp Clin Psychopharmacol.

[CR57] Steinberg ML, Williams JM, Ziedonis DM (2004). Financial implications of cigarette smoking among individuals with schizophrenia. Tob Control.

[CR58] Wilhelm CJ, Mitchell SH (2009). Strain differences in delay discounting using inbred rats. Genes Brain Behav.

[CR59] Wing VC, Moss TG, Rabin RA, George TP (2012). Effects of cigarette smoking status on delay discounting in schizophrenia and healthy controls. Addict Behav.

[CR60] Yoon JH, Higgins ST, Heil SH (2007). Delay discounting predicts postpartum relapse to cigarette smoking among pregnant women. Exp Clin Psychopharmacol.

